# The Social Gradient in Tobacco Use Does Not Generalize to Low-Income Urban Communities in India: Findings From a Census Survey

**DOI:** 10.1093/ntr/ntw214

**Published:** 2016-08-23

**Authors:** Bidyut K Sarkar, Lion Shahab, Monika Arora, Jasjit S Ahluwalia, K Srinath Reddy, Robert West

**Affiliations:** 1 Centre for Tobacco Control and Health Promotion, Public Health Foundation of India, New Delhi, India; 2 Department of Epidemiology and Public Health, University College London, London, UK; 3 School of Public Health, Rutgers School of Public Health, Piscataway, NJ

## Abstract

**Introduction:**

The existence of a social gradient in tobacco use has been clearly established in a number of countries with people with lower socioeconomic status being more likely to use tobacco. It is not clear how far this gradient is evident within severely deprived communities. This study assessed the association between occupation as a marker of socioeconomic status and use of smoked and smokeless tobacco within “slum” areas of Delhi, India.

**Methods:**

A census survey of 11 888 households, comprising 30 655 adults from 28 low-income communities (14 government-authorized and 14 unauthorized settlements called “Jhuggi-Jhopri/JJ” clusters) was conducted in 2012. The survey assessed age, sex, household size, occupational group, and current tobacco use. Independent associations with tobacco use were conducted using complex samples regression analysis, stratified by gender.

**Results:**

A quarter of participants (24.3%, 95% confidence interval [CI] 21.5–27.5) used any tobacco. Slightly more people used smoked (14.6%, 95% CI 12.9–16.3) than smokeless (12.6%, 95% CI 10.7–14.8) tobacco, with a small minority being dual users (2.7%, 95% CI 2.1–3.5). Prevalence of any tobacco use was highest in unskilled (45.13%, 95% CI 42.4–47.9) and skilled (46.2%, 95% CI 41.1–51.4) manual occupations and lower in nonmanual (30.3%, 95% CI 26.2–34.7) occupations and those who were unemployed (29.0%, 95% CI 25.3–33.0). This was confirmed in adjusted analysis in men but associations were more complex in women.

**Conclusions:**

Use of smoked and smokeless tobacco in low-income urban communities in India has a complex association with occupational status with both nonmanual occupation and unemployment being associated with lower prevalence of smoked and smokeless tobacco in men.

**Implications:**

Tobacco use in high-income countries shows a strong inverse relationship with social grade, income, and deprivation such that use is much more common among those who can least afford it. This study is the first to look at this social gradient in the context of low-income communities in India, finding that both unemployment and nonmanual occupation were associated with lower rates of tobacco use in men. The data present a challenge to existing explanations of the social gradient, requiring further consideration of the conditions under which affordability may work to reduce health inequalities arising from tobacco use.

## Introduction

Tobacco use is more prevalent in people with lower socioeconomic status in most countries.^1^ It is a major source of health inequality.^2^ However, it is not clear how generalizable this gradient is globally. For example, it is not clear how far the gradient follows the same pattern within deprived communities in low- or middle-income countries and with smoked, as well as smokeless tobacco. Neither is it clear whether a similar pattern holds for employment status (being in or out of work) and occupational status (grade of the work). Gathering data on this is important to understanding what underlies the phenomenon and developing appropriately targeted interventions. We explored this in a large survey in low-income urban communities in India.

Tobacco use in India contributes substantially to the global burden of disease. Of the 6 million or so annual deaths from tobacco, nearly 1 million are in India^3^ and related health-care costs in that country amount to billions of dollars a year.^4^ The Indian tobacco market is complex^5^ with a wide variety of both smoked and smokeless tobacco products that may show different associations with socioeconomic variables.

Although it has been argued that conventional tobacco control measures (eg, taxation and smoking bans) should be effective in India,^6^ because of weak regulatory and law enforcement mechanisms such strategies may not work, as well as elsewhere.^7^ It has been proposed that increasing the financial cost of smoking through tax increases should disproportionately benefit the most disadvantaged tobacco users because of higher price elasticity.^8^ However, this depends on the social gradient being essentially monotonic and does not take account of complexities around illicit supply and cross border traffic. Effective, practicable and affordable interventions to curb the tobacco epidemic are urgently needed.^9^ Crucially, interventions in countries such as India need to take account of equity (need to reduce relative economic, social and health disadvantage), and this requires a better understanding of the sociodemographic factors that might influence tobacco use.

“Slum” areas in Delhi represent potentially useful locations to undertake studies to assess the impact of socioeconomic factors on tobacco use. Residents can all be expected to be experiencing some degree of housing and economic stress, and yet there should be some variation in this that could allow for links with tobacco use to emerge. The slums are very densely populated and population mobility is relatively low. Many of the residents have come from rural communities in India covering a wide geographical area. Undertaking research in these areas is challenging but structures have been set up to undertake this work, with relationships established with nongovernmental organizations.

To assess how far socioeconomic factors influence tobacco use would generalize to low-income communities in a middle income country, we examined associations between socioeconomic status as assessed by occupational group with smoked and smokeless tobacco use in slum areas of Delhi.

## Methods

### Study Setting and Design

The study population comprised 28 urban slum settlements in Delhi; 14 were government authorized “resettlement colonies” and 14 were unauthorized settlements called “Jhuggi-Jhopri” or JJ clusters. The authorized resettlement colonies have government authorized electricity and water supply and the houses are mostly permanent brick structures whereas the JJ clusters are semi-permanent houses without authorized electricity or water supply. Residents of JJ clusters are poorer than those of resettlement colonies. The 28 communities were selected for an earlier research study on youths aged 10–19 years from a list of registered resettlement colonies (*n* = 44) procured from the Municipal Corporation of Delhi and the Department of Health and Family Welfare. The survey took place from January to August 2012.

Four field researchers collected responses with support from an NGO which had worked in these communities and had established a rapport with community leaders and residents. Each household of the selected administrative block of the urban slum area was visited by a member of the survey team. The questionnaire was administered during a face-to-face interview with an adult from the household. Where feasible, a repeat visit was made on the next day to houses that were locked or where household members were unavailable.

### Participants

These low-income communities had been selected for a previous tobacco study conducted among youths aged 10–19 years in 2009,^10^ therefore only adults aged 23 or above were eligible to be selected for this census to avoid contamination with the previous study. A total of 30 655 residents in 11 888 households were surveyed.

### Measures

Participants were first asked to report all the adult residents currently living in the household. Then, data for tobacco consumption for each eligible household member including the respondent were collected. Participants were asked whether they or other household members smoke bidi, cigarettes or hukkah; whether they chewed khaini, tobacco gutka or paan with zarda or used any other form of tobacco (cigar, gul manjan, a tobacco toothpaste or surti). Based on responses, individuals were classified as “smokers,” “chewers,” or “dual users.” Sociodemographic information regarding the age, gender and occupation of each eligible adult was recorded. The type of community in which they resided was recorded (Resettlement or JJ).

### Analysis

We used complex samples, single and multivariable logistic regression analyses to predict tobacco use status, stratified by gender (any tobacco, smoked vs. smokeless and single vs. dual forms).

## Results

The mean (*SEM*) age of the sample was 39.1 years (0.24); 53.9% (*N* = 16 509) were male; mean (*SEM*) household size was 3.3 (0.08); 41.9% (*N* = 12 846) lived in JJ clusters. Occupational groups were: 12.9% (*N* = 3958) unskilled manual; 9.2% (*N* = 2811) skilled manual; 31.3% (*N* = 9573) skilled nonmanual; 34.0% (*N* = 10 414) housewife; 10.5% (*N* = 3215) unemployed; 2.0% (*N* = 626) other.

Nearly a quarter of participants surveyed (24.3%, 95% confidence interval [CI] 21.5–27.5) were using tobacco of some kind. Slightly more people used smoked (14.6%, 95% CI 12.9–16.3) than smokeless (12.6%, 95% CI 10.7–14.8) products; a small proportion were dual users of smoked and smokeless products (2.7%, 95% CI 2.1–3.5). Bidis were by far the most prevalent smoked tobacco product (13.0%, 95% CI 11.4–14.7%), followed by cigarettes (3.7%, 95% CI 3.1–4.5) and hookahs (0.2%, 95% CI 0.1–0.3). There was almost no cigar use and a small proportion (2.3%, 95% CI 1.9–2.8) used more than one smoked tobacco product. Khaini was the most popular smokeless tobacco product (7.5%, 95% CI 6.1–9.2), and gutkha was the second most popular product (5.3%, 4.5–6.1). Few respondents used zarda (1.2%, 95% CI 0.9–1.6), paan with tobacco (1.2%, 95% CI 0.9–1.6), or gul/surti (0.1%, 95% CI 0.0–0.1). A small proportion used more than one smokeless tobacco product (2.4%, 95% CI 2.0–3.0).

**Figure 1.**
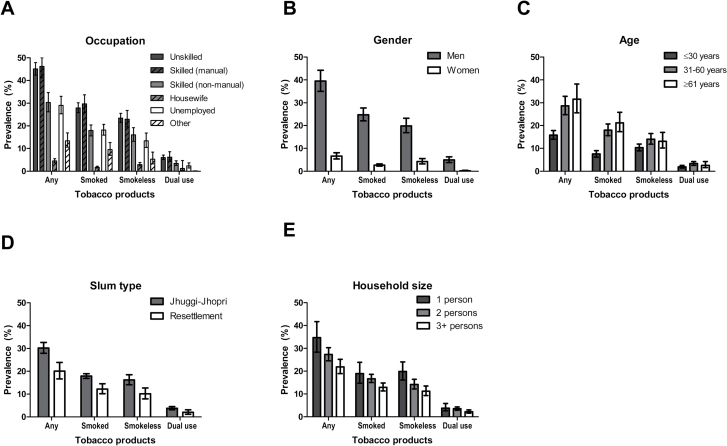
Prevalence of different forms of tobacco use by sociodemographic characteristics.

The distribution of tobacco use by sociodemographic variables is provided in Figure 1. Prevalence of any tobacco use was highest in skilled manual occupations (46.2%, 95% CI 41.1–51.4), followed by unskilled (45.13%, 95% CI 42.4–47.9) and skilled nonmanual occupations (30.3%, 95% CI 26.2–34.7), those who were unemployed (29.0%, 95% CI 25.3–33.0) and was lowest among those classified as “Other” (13.0%, 95% CI 10.4–16.1) and as housewives (4.3%, 95% CI 3.5–5.3, Figure 1A).

As tobacco use was far more prevalent in men than women (Figure 1B), further analyses were stratified by gender. Table 1 and Figure 1, C–E show that for both men and women any tobacco use was associated with being older, living in a JJ cluster and having a smaller household size. In terms of occupation, tobacco use in men was higher in unskilled and skilled manual occupations, but similar to those who were in skilled nonmanual occupations compared with those who were unemployed. Among women, tobacco use was higher for those in unskilled manual occupations and lower for housewives compared with those who were unemployed but there were no other differences. Findings did not materially alter in a fully adjusted model. Controlling for all other variables, smoked as compared with smokeless tobacco use was also associated with being older in men and in women with being from a larger household, as was dual versus single use.

**Table 1.** Univariable and Multivariable Associations With Type of Tobacco Use, Stratified by Gender

	Any vs. no tobacco use		Smoked vs. smokeless tobacco use^a^		Dual vs. single use^b^	
	Model 1; odds ratio (95% CI)	Model 2; adjusted odds ratio (95% CI)	Model 1; odds ratio (95% CI)	Model 2; adjusted odds ratio (95% CI)	Model 1; odds ratio (95% CI)	Model 2; adjusted odds ratio (95% CI)
Men	*N* = 16 509	*N* = 16 416	*N* = 5725	*N* = 5708	*N* = 6526	*N* = 6507
Age^c^	1.03 (1.02–1.03)*	1.03 (1.02–1.04)*	1.03 (1.02–1.05)*	1.03 (1.02–1.05)*	0.99 (0.99–1.00)	1.00 (0.99–1.00)
Household size^d^	0.83 (0.78–0.88)*	0.87 (0.83–0.91)*	0.99 (0.94–1.04)	0.97 (0.92–1.02)	0.91 (0.82–1.00)***	0.91 (0.82–1.00)
Occupation	*	*				
Unemployed	1	1	1	1	1	1
Unskilled	1.61 (1.35–1.92)	1.60 (1.35–1.90)	0.77 (0.58–1.01)	1.02 (0.72–1.45)	1.62 (1.01–2.60)	1.40 (0.89–2.20)
Skilled (manual)	1.25 (1.01–1.54)	1.45 (1.18–1.78)	0.80 (0.61–1.05)	1.16 (0.85–1.58)	1.52 (1.02–2.27)	1.37 (0.91–2.04)
Skilled (nonmanual)	0.71 (0.61–0.82)	0.86 (0.70–1.06)	0.67 (0.51–0.88)	0.92 (0.66–1.27)	1.30 (0.87–1.94)	1.18 (0.78–1.79)
Other	0.21 (0.15–0.30)	0.25 (0.18–0.35)	1.09 (0.60–1.97)	1.02 (0.52–1.99)	1.14 (0.36–3.61)	1.12 (0.36–3.53)
Housewife	—	—	—	—	—	—
JJ cluster^e^	1.97 (1.50–2.60)*	1.74 (1.35–2.25)*	0.90 (0.72–1.11)	0.89 (0.70–1.13)	1.31 (0.88–1.96)	1.22 (0.83–1.79)
Women	*N* = 14 145	*N* = 14 046	*N* = 906	*N* = 899	*N* = 936	*N* = 929
Age^c^	1.05 (1.04–1.06)*	1.05 (1.04–1.06)*	1.01 (1.00–1.02)	1.01 (1.00–1.02)	1.01 (0.98–1.04)	1.02 (1.00–1.04)
Household size^d^	0.89 (0.83–0.94)*	0.90 (0.84–0.97)**	1.12 (1.00–1.26)	1.12 (1.00–1.26)***	1.29 (0.98–1.70)	1.35 (1.04–1.76)***
Occupation	***	**				
Unemployed	1	1	1	1	1	1
Unskilled	1.38 (1.06–1.86)	1.38 (1.05–1.83)	0.95 (0.46–1.93)	1.08 (0.51–2.26)	1.71 (0.45–6.54)	2.05 (0.52–8.08)
Skilled (manual)	1.10 (0.47–2.61)	1.59 (0.56–4.47)	1.08 (0.18–6.36)	1.35 (0.24–7.57)	10.1 (0.88–115.1)	11.4 (1.31–99.8)
Skilled (nonmanual)	0.78 (0.59–0.97)	0.90 (0.66–1.23)	0.97 (0.57–1.66)	1.06 (0.61–1.83)	1.15 (0.39–3.42)	1.49 (0.47–4.71)
Other	1.01 (0.43–2.36)	1.72 (0.76–3.93)	1.44 (0.44–4.72)	1.91 (0.61–5.99)	2.93 (0.28–30.5)	7.63 (0.69–84.4)
Housewife	0.31 (0.25–0.37)	0.40 (0.31–0.51)	0.79 (0.57–1.11)	0.87 (0.60–1.26)	0.96 (0.35–2.67)	1.19 (0.44–3.18)
JJ cluster^e^	1.65 (1.11–2.44)***	1.67 (1.14–2.44)***	0.92 (0.55–1.52)	1.00 (0.59–1.68)	1.85 (0.79–4.35)	2.02 (0.87–4.71)

CI = confidence interval; JJ = Jhuggi-Jhopri. Model 1: All variables entered separately (univariable model); Model 2: All variables entered together (multivariable model).

^a^Excludes dual users.

^b^Refers to use of combustible or noncombustible products or both.

^c^Change per year.

^d^Change per householder.

^e^vs. resettlement colony.

*p < .001; **p < .01; ***p < .05.

## Discussion

The fact that unemployment was associated with lower smoking rates than being in a manual occupation suggests that work-related factors other than educational level and financial stress may influence tobacco use in some communities. One possible explanation is that unemployment in these communities results in such a low income that tobacco is not affordable. However, research in China has found that poverty was associated with lower consumption and purchase of cheaper products rather than not using tobacco at all.^11^ In India, tobacco products can be extremely cheap and it seems implausible that users would necessarily stop altogether and restart again as they went into and out of unemployment rather than changing the amount they use. The data present a challenge to existing explanations of the social gradient in tobacco and merit further study. They also require consideration of the conditions under which tax increases may work to reduce health inequalities arising from tobacco use, and that this may be gender-specific.

This study had several limitations. The prevalence estimates were based on reports by the head of household. Prevalence surveys based on self-report are considered reasonably accurate but there may be additional inaccuracy resulting from surrogate reporting. This merits further research. Secondly, data could not be collected from a number of households; reasons included locked households, respondents being unavailable at the time of visit or households refusing to participate. However, this proportion was small (<10%) and so is unlikely to have had a substantial impact on the findings. Thirdly, the survey did not cover people under the age of 23 which limits conclusions that can be drawn regarding youth tobacco use in India. Fourthly, it is not clear how far the findings generalize to other cities in India or indeed to rural areas. Lastly, we used occupation as an index of socioeconomic status which does not necessarily map one-to-one onto other deprivation measures such as education and may have a more complex relationship than a simpler gradated measure such as income. Nevertheless, occupation is commonly used as a measure to assess wealth and status,^12^ and the pattern of findings does raise important issues concerning the link between socioeconomic status and tobacco use that require further study.

This study also has a number of strengths. The large sample size allowed relatively precise estimation of parameters. It employed a rigorous methodology to identify participants, and provided near complete coverage of disparate low-income communities in Delhi. It also covered all forms of tobacco use.

## Funding

This work was supported by a Wellcome Trust Capacity Strengthening Strategic Award to Public Health Foundation of India and a consortium of UK Universities (WTP grant reference no: 6936) with additional support from Cancer Research UK and the UK’s National Centre for Smoking Cessation and Training.

## Declaration of Interests

*BKS was funded by a UKC-Wellcome trust capacity strengthening strategic award to PHFI and has no conflict of interest to declare. MA, JSA, and KSR have no conflict of interest to declare. LS is a HEFCE-funded member of staff at University College London. He has received an honorarium for a talk, an unrestricted research grant and travel expenses to attend meetings and workshops from Pfizer, a pharmaceutical company that makes smoking cessation products, and has acted as paid reviewer for grant awarding bodies and as a paid consultant for health care companies. Other research has been funded by the government, a community-interested company (National Centre for Smoking Cessation) and charitable sources. He has never received personal fees or research funding of any kind from alcohol, electronic cigarette or tobacco companies. RW undertakes consultancy and research for and receives travel funds and hospitality from manufacturers of medications for smoking cessation.*
